# PD-L1 Expression in Paired Samples of Rectal Cancer

**DOI:** 10.3390/cancers16142606

**Published:** 2024-07-21

**Authors:** Mina Coussement, Roberta Fazio, Alessandro Audisio, Reem El Khoury, Fatima-Zahra Abbassi, Irene Assaf, Chiara Conti, Chiara Gallio, Nada Benhima, Giacomo Bregni, Paraskevas Gkolfakis, Valentina Spagnolo, Geraldine Anthoine, Gabriel Liberale, Luigi Moretti, Philippe Martinive, Alain Hendlisz, Pieter Demetter, Francesco Sclafani

**Affiliations:** 1 Institut Jules Bordet, Université Libre de Bruxelles (ULB), Hôpital Universitaire de Bruxelles (HUB), 1070 Brussels, Belgium; mina.coussement@ulb.be (M.C.); roberta.fazio@cancercenter.humanitas.it (R.F.); valentina.spagnolo@ulb.be (V.S.);; 2 Cerba Path, Division CMP, 1070 Brussels, Belgium; 3 Laboratory for Experimental Gastroenterology, Université Libre de Bruxelles (ULB), 1070 Brussels, Belgium

**Keywords:** PD-L1, tumour proportion score (TPS), immune cell score (ICS), combined positive score (CPS), immune checkpoint inhibitors, radiotherapy, rectal cancer

## Abstract

**Simple Summary:**

There is an increased interest for the investigation of immunotherapy and immune-related biomarkers in rectal cancer. We retrospectively analysed the expression of the programmed death-ligand 1 (PD-L1) in diagnostic biopsies and resection samples from a cohort of 83 rectal cancer patients. Using three different methods for the analysis of PD-L1, we found that the expression of this biomarker was lower in resection samples than in diagnostic biopsies. Also, we observed that higher levels of PD-L1 in resection specimens were associated with better survival outcomes. The results of our study contribute to the knowledge of PD-L1 expression in rectal cancer, having the potential to inform the design of future immunotherapy trials in this setting.

**Abstract:**

Immune checkpoint inhibitors and immune-related biomarkers are increasingly investigated in rectal cancer (RC). We retrospectively analysed PD-L1 expression in diagnostic biopsy and resection samples from RC patients treated at our centre between 2000 and 2020. PD-L1 immunostaining (22C3 clone) was evaluated according to tumour proportion (TPS), immune cell (ICS), and the combined positive score (CPS). Eighty-three patients were included. At diagnosis, PD-L1 expression ≥1%/≥5% was observed in 15.4%/0%, 80.7%/37.4%, and 69.2%/25.6% of patients based on TPS, ICS, and CPS, respectively. At surgery, the respective figures were 4.6%/1.5%, 60.2%/32.5%, and 50.7%/26.2%. Using the 1% cut-off and regardless of the scoring system, PD-L1 was less expressed in surgery than biopsy samples (*p* ≤ 0.04). In paired specimens, PD-L1-ICS reduction was especially observed following neoadjuvant long-course (chemo)radiotherapy (*p* = 0.03). PD-L1-ICS of ≥5% in surgical samples (HR: 0.17; *p* = 0.02), and a biopsy-to-surgery increase in PD-L1-ICS (HR: 0.19; *p* = 0.04) was predictive for longer disease-free survival, while the PD-L1-ICS of either ≥1% (HR 0.28; *p* = 0.04) or ≥5% (HR 0.19; *p* = 0.03) in surgical samples and the biopsy-to-surgery increase in PD-L1-ICS (HR: 0.20; *p* = 0.04) were associated with better overall survival. Our study suggests that PD-L1 expression in RC is largely reflective of immune cell infiltration, and its presence/increase in surgical samples predicts better outcomes.

## 1. Introduction

Rectal cancer is the 8th most common malignancy and the 10th leading cause of cancer-related mortality [1]. In 2022, 729,702 individuals were diagnosed with rectal cancer worldwide, and 343,761 died of this disease. Notably, while rectal cancer accounts for approximately one third of all colorectal tumours, this is the most common type of bowel cancer in young adults. According to the American Cancer Society, new rectal cancer cases among people younger than 55 years doubled from 11% in 1995 to 20% in 2019 [2].

The management of locally advanced rectal cancer has recently evolved with the development of a number of different, risk-adapted, treatment options [3,4,5,6,7,8,9]. Nevertheless, this still largely relies on the variable use and combination of chemotherapy, radiotherapy, and surgery [10,11]. In single-arm phase II trials of colon and rectal cancers, neoadjuvant immune checkpoint inhibitors alone have yielded impressive rates of pathological and/or clinical complete responses [12,13,14,15,16,17,18,19]. These results, however, were achieved in a small group of patients with microsatellite instable (MSI-H) or mismatch repair deficient (dMMR) tumours (approximately 3% of all rectal cancers) [20]. Efficacy results in the larger group of patients with microsatellite stable (MSS) or mismatch repair proficient (pMMR) tumours are far less interesting [14,19].

Overcoming the inherent resistance of pMMR/MSS tumours, which are generally characterised by a lower tumour mutation burden (TMB) and lymphocyte infiltration as compared with their dMMR/MSI-H counterparts, has now become one of the key unmet needs of clinical research in this field [21,22]. Several different approaches have been tested, including the combination of immune checkpoint inhibitors with radiotherapy and chemotherapy, with variable results [23,24]. Both radiotherapy and chemotherapy can indeed modulate the tumour microenvironment and eventually enhance the host immune response (and so the therapeutic potential of immune checkpoint inhibitors) [25,26,27]. Yet, it is not known how to best combine immunotherapy with the standard therapies that are commonly used for the rectal cancer multimodal treatment.

Programmed death ligand 1 (PD-L1) is an immunotherapeutic target, which is routinely tested in many cancer types to select patients who are most likely to benefit from immune checkpoint inhibitors [28,29,30,31,32]. Both in dMMR/MSI-H and pMMR/MSS colorectal tumours, PD-L1 does not appear to be a valid predictive biomarker [33]. Nevertheless, based on its key role in cancer immune evasion, it can still provide useful information regarding the effects of the chemotherapy- and/or radiotherapy-induced immunomodulation, and inform the optimal development of immunotherapy-based, therapeutic strategies for pMMR/MSS tumours [23,24]. At this stage, studies regarding the expression of PD-L1 in rectal cancer and its variations following neoadjuvant therapy are limited overall, and their results are largely inconsistent [34,35,36,37,38,39,40,41,42,43,44,45,46,47,48,49,50,51].

We therefore sought to contribute to the understanding of the potential value of this biomarker by analysing a series of rectal cancer patients who were treated according to standard practise at our institution.

## 2. Materials and Methods

### 2.1. Study Design

This is a single-centre, retrospective study including consecutive patients who had undergone surgery for rectal adenocarcinoma at the Institut Jules Bordet (Brussels, Belgium) between January 2000 and January 2020. The eligibility criteria were as follows: distal border of the tumour within 15 cm of the anal verge as determined by either pelvic MRI or endoscopy, and availability of tumour tissue material from both the pre-treatment diagnostic biopsy and the post-treatment (if any) surgical specimen. Patients were included irrespective of their clinical tumour stage or the type of preoperative treatment received (if any). Patients who had received more than one treatment (due to tumour progression) prior to surgical resection of the primary tumour were excluded, as were those who had surgery for recurrent tumours.

Clinical data were collected from the electronic patient record system and entered into a study-specific database. These included demographics, clinico-pathological features at baseline, treatment details, pathological features at surgery, pathological response to preoperative treatment, and long-term treatment and survival outcomes.

The study was approved by the Ethics Committee at the Institut Jules Bordet (CE3251). Given the retrospective design, no specific consent was obtained from study patients.

### 2.2. Immunohistochemistry

Archived tumour tissue from the pre-treatment diagnostic biopsy and the post-treatment (if any) surgical specimen were collected for each study patient. Tumour blocks were cut into 4 µm slices and processed via immunohistochemistry. PD-L1 analysis was carried out using the antibody clone 22C3 (pharmaDX kit, Agilent Technologies, Santa Clara, CA, USA). Human placenta was used as a positive control.

PD-L1 expression was assessed by an experienced gastrointestinal pathologist who was blinded to the clinical data. Expression was evaluated according to three different scoring systems: the tumour proportion score (TPS), the immune cell score (ICS), and the combined positive score (CPS). The TPS represents the percentage of viable tumour cells showing partial or complete membrane staining at any intensity [52]. The ICS is the percentage of tumour area covered by PD-L1 positive immune cells [53]. The CPS is defined as the number of PD-L1 stained cells (among tumour cells, lymphocytes, and macrophages), divided by the total number of viable tumour cells, multiplied by 100 [54]. For the three different scoring systems, PD-L1 expression was rounded to the nearest of the following percentage values: 0, 1, 5, 10, 25, 50.

### 2.3. Statistical Analysis

The primary objective of this study was to analyse the expression of PD-L1 according to three different scores in tumour biopsy and resection samples. Secondary objectives included the following: the analysis of the association between PD-L1 expression and baseline clinico-pathological characteristics, and the analysis of the changes in PD-L1 expression between biopsy and resection samples in the entire study population and by type of preoperative treatment. Exploratory survival analyses by PD-L1 expression in both biopsy and resection specimens and by variations in PD-L1 expression in paired specimens were also planned.

The differences in categorical variables were compared using the two-sided Fisher’s exact test. The Cox proportional hazards model was used for univariable/multivariable analyses and survival analyses. Variables with a *p*-value of ≤0.1 in the univariable model were entered in the multivariable model. Disease-free survival (DFS) was defined as the time from rectal surgery to tumour recurrence or death from any cause, while overall survival (OS) was defined as the time from rectal surgery to death from any cause. Patients with stage IV tumours at the time of the diagnostic biopsy or surgical resection were excluded from all the survival analyses. Survival was estimated with the Kaplan–Meier method, while the reverse Kaplan–Meier method was used to calculate the median follow-up. *p* values were considered as significant if <0.05. All statistical analyses were performed with R Statistical Software (v4.4.0; R Foundation, Vienna, Austria).

## 3. Results

### 3.1. Baseline Characteristics

Eighty-three patients met the eligibility criteria and were included in the study. Detailed information on the study population is provided in Table 1. The median age at diagnosis was 63 years (range 30–98), with a relatively even distribution between males and females. The majority of patients (69.1%) presented with stage III tumours, while 11 (13.3%) had metastatic disease at diagnosis. Preoperative therapy was administered to most patients (88%), this consisting of radiotherapy (either alone or with concurrent fluoropyrimidines) in 66.3%, total neoadjuvant therapy (TNT, including the administration of both radiotherapy and systemic chemotherapy before surgery) in 19.3%, and chemotherapy alone in 2.4% of cases. Regarding radiotherapy modalities, long-course radiotherapy (with [LC-CRT] or without [LC-RT] concurrent chemotherapy) was delivered to 68.7%, while short-course radiotherapy (SCRT) was delivered to 16.9% of patients.

### 3.2. PD-L1 Expression in Diagnostic Biopsies and Surgical Specimens

In diagnostic biopsy samples, PD-L1 expressions of ≥1% and ≥5%, respectively, were found in 15.4% and 0% of patients according to the TPS, in 80.7% and 37.4% according to the ICS, and in 69.2% and 25.6% according to the CPS. Baseline clinico-pathological characteristics by PD-L1 expression are outlined in Supplementary Tables S1 and S2. No statistically significant association was identified between PD-L1 expression (regardless of the scoring system and cut-off values) and the age, sex, tumour differentiation, tumour location, cT stage, and cN stage.

**Figure 1:**
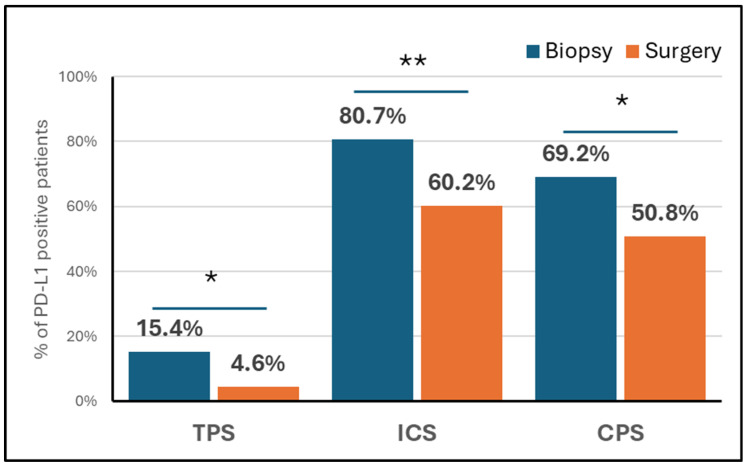
PD-L1 expression ≥1% according to TPS, IC and CPS in biopsy and surgical specimens. * *p* value < 0.05; ** *p* value < 0.01. Abbreviations: CPS, combined positive score; ICS, immune-cell score; TPS, tumour proportion score.

Pathological characteristics at surgery are reported in Supplementary Table S3. Most patients (83.1%) had an R0 resection, while 8.4% had a pathological complete response. In surgical specimens, PD-L1 expressions of ≥1% and ≥5%, respectively, were detected in 4.6% and 1.5% of cases based on the TPS, 60.2% and 32.5% based on the ICS, and 50.7% and 26.2% based on the CPS. When using 1% as the cut-off to distinguish positive versus negative cases, the proportion of PD-L1-positive tumours decreased from the biopsy to the resection samples across all scoring systems. This difference was statistically significant for the TPS (*p* = 0.036), ICS (*p* = 0.004), and CPS (*p* = 0.024), as illustrated in Figure 1. However, no significant difference was noted between biopsy and surgical samples when the 5% cut-off was used (Supplementary Figure S1).

### 3.3. Changes in PD-L1 Expression in Paired Specimens

For this analysis, a change in PD-L1 expression was defined as any variation in expression according to the rounded percentage values of 0, 1, 5, 10, 25, and 50. The proportions of patients who had reduced, stable, and increased PD-L1 expression, respectively, between paired biopsy and resection samples were 15.9%, 79.3%, and 4.8% by the TPS, 41%, 30.1%, and 28.9% by the ICS, and 34.9%, 39.7%, and 25.4% by the CPS. Among all the treatment-related variables analysed, only LC-(C)RT (as compared with SCRT) was associated with a statistically significant reduction in PD-L1 expression in surgical specimens (49.1% vs. 14.3%, *p* = 0.03), and this was limited to the analysis by the ICS (Table 2).

### 3.4. PD-L1 Expression/Change and Survival Outcomes

At the time of the analysis, 31 patients (34%) had died. The median follow-up duration for the entire study population was 78 months (95% confidence intervals [CI]: 69–93). In univariable analyses for DFS, a PD-L1 expression of ≥5% in surgical samples by the CPS (HR: 0.32; 95% CI: 0.1–1.1; *p* = 0.067) and ICS (HR: 0.34; 95% CI: 0.13–0.89; *p* = 0.029) and a biopsy-to-surgery increase in PD-L1 by the ICS (HR: 0.44; 95% CI: 0.17–1.2; *p* = 0.095) were found to be statistically significant (Supplementary Table S4). After multivariable analyses, only a PD-L1 expression of ≥5 in surgical samples by the ICS (HR: 0.17; 95% CI: 0.04–0.77; *p* = 0.02) and biopsy-to-surgery increase in PD-L1 by the ICS (HR: 0.19; 95% CI: 0.04–0.89; *p* = 0.04) remained statistically significant (along with other conventional prognostic factors). Detailed results are available in Supplementary Tables S4 and S5.

**Figure 2:**
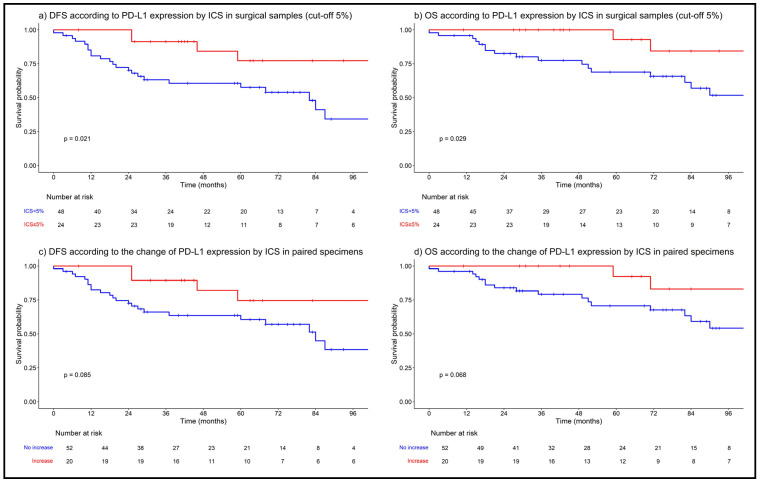
Disease-free and overall survival according to PD-L1 by ICS in surgical samples (**a**,**b**), and according to change in PD-L1 expression by ICS in paired specimens (**c**,**d**). Abbreviations: ICS, immune-cell score; DFS, disease-free survival; OS, overall survival.

In univariable analyses for OS, PD-L1 expressions in surgical samples of ≥1% (HR: 0.46; 95% CI: 0.19–1.1; *p* = 0.084) and ≥5% (HR: 0.28; 95% CI: 0.08–0.95; *p* = 0.042) by the ICS and of ≥5 by the CPS (HR: 0.14; 95% CI: 0.02–1.1; *p* = 0.059) and a biopsy-to-surgery increase in PD-L1 by the ICS (HR: 0.34; 95% CI: 0.099–1.2; *p* = 0.082) were found to be statistically significant (Supplementary Table S6). After multivariable analyses, only PD-L1 expressions in surgical samples of ≥1% (HR 0.28; 95% CI: 0.08–0.97, *p* = 0.04) and of ≥5% (HR 0.19; 95% CI: 0.04–0.88, *p* = 0.03) by the ICS and a biopsy-to-surgery increase in PD-L1 by the ICS (HR: 0.20; 95% CI: 0.04–0.93; *p* = 0.04) were significantly associated with better OS (along with other conventional prognostic factors) (Supplementary Table S7). Kaplan–Meier curves for DFS and OS by different PD-L1 scores are shown in Figure 2 and Supplementary Figures S2–S5.

## 4. Discussion

In this study, we analysed PD-L1 expression in paired tumour specimens from a cohort of rectal cancer patients treated according to standard practise at a tertiary cancer centre. We found that the expression of this biomarker varied based on the staining score. Most patients were PD-L1-positive according to the ICS and CPS, while PD-L1-negative according to the TPS. PD-L1 was more frequently expressed in the diagnostic biopsies than in the resection specimens, preoperative LC-(C)CRT being the only factor associated with a biopsy-to-surgery PD-L1 reduction based on the ICS. Finally, among patients treated with curative intent, PD-L1 expression or increase in the ICS in surgical samples were independent prognostic factors.

The PD-1/PD-L1 pathway is the target of most immune checkpoint inhibitors that have been approved for the treatment of solid tumours. The expression of PD-L1 as well as its association with treatment outcome have extensively been studied, and in some cases, the use of immune checkpoint inhibition is restricted to PD-L1-positive patients [28,29,30,31,32]. However, given that immunotherapy is not a standard treatment option for rectal cancer patients (with the only exception of those with metastatic dMMR/MSI-H tumours, for whom PD-L1 is not a predictive factor anyway), the evidence-building process regarding the frequency, dynamics, and predictive/prognostic value of this biomarker in this disease has lagged behind [55].

The first point that has not yet been adequately addressed concerns the optimal assessment method. With the intention to shed light into this topic, we tested PD-L1 according to the three most common immunostaining scores. We observed a substantial variation in expression, with most patients being classified as positive (regardless of the intensity of expression) based on the CPS and ICS, and as negative based on the TPS. Such an inter-score variation is not entirely unexpected, considering the different cellular components that contribute to the definition of PD-L1 positivity (tumour cells only for the TPS, immune cells only for the ICS, and the combination of both for the CPS). Bearing in mind the wide range of PD-L1 expression reported in the literature (ranging between 0% and 97% in tumour cells and between 15% and 97% in immune cells) [34,35,36,37,38,41,42,43,44,47,48,49,51], our findings are supported by several series where PD-L1 expression was shown to be higher in immune cells than in tumour cells [34,36,37,39,42,43,47,48,49,51] (Supplementary Table S8). Nevertheless, it should be acknowledged that contrasting results were reported by the other two studies that analysed PD-L1 expression using the three different scores. Huemer et al. did not observe any significant inter-score variability, the expression of PD-L1 being invariably high [47], while Feng et al. showed a lower mean PD-L1 value with the IC than with the CPS or the TPS [48]. This inconsistency could be explained by several factors. First, not all studies used the same criteria for the definitions of the TPS, ICS, and CPS as recently established, and certain nuances in the interpretation of the scoring results might have influenced the overall readouts [34,35,36,37,41,46,50]. Second, we used cut-offs of ≥1% [38,47,48] and ≥ 5% [42,44,56], whereas different cut-offs including mean/median values were considered in other studies for the dichotomisation between positive and negative cases [34,39,41,46,49,50,57]. Third, analytical factors such as the choice of the anti-PD-L1 antibody for the immunohistochemical staining may partly explain the variability in PD-L1 expression. We used 22C3 based on its high sensitivity [58], but other clones showing a good agreement for the detection of PD-L1 expression on tumour cells across several cancer types are available [59]. Notably, in a study by Li et al., SP263 appeared to be the most sensitive clone for PD-L1 detection on both tumour and immune cells from colorectal cancer [60].

An interesting finding of our analysis was the reduced expression of PD-L1 from biopsy to surgical samples. Data from rectal cancer studies looking at the dynamics of PD-L1 expression under preoperative treatment are not unequivocal. Our results are in line with those from Huemer et al. (decreased expression regardless of the scoring system) [47], and only partially consistent with those from Hecht et al. (decreased expression only in immune cells; increased expression in tumour cells) [36]. On the other hand, in a number of other series, PD-L1 expression in either immune cells [37,42,43,48], tumour cells [41,44,61], or both [51] has been reported to increase from the diagnostic biopsies to the surgical resection specimens. It is believed that both radiotherapy and chemotherapy can have immunomodulatory effects by modifying the tumour microenvironment. In particular, as radiotherapy is known to induce immunogenic cell death by releasing tumour-associated antigens and danger signals (DAMPs) which, in turn, cause cytokine release (including Interferon gamma, a positive regulator of PD-L1), the recruitment of dendritic cells, and the proliferation of CD8+ T cells, we would normally expect an increased expression of this biomarker after irradiation [26,62]. Nevertheless, it has also been shown that the expression of PD-L1 following radiotherapy may change over time. In a preclinical study using mouse models, the peak of PD-L1 expression occurred 3 days after the last dose of radiotherapy, while it decreased significantly after 7 days [62]. In our study, the potential impact of the radiotherapy-to-surgery interval on the dynamics of PD-L1 expression was evaluated. Although we did not find any difference between patients who had undergone early versus delayed surgery, we cannot rule out that the cut-off value used for this analysis (i.e., median value of 2 months) might have precluded the detection of early radiotherapy-induced effects. In contrast, we observed an association between the variation in PD-L1 expression in paired specimens and the type of preoperative radiotherapy, SCRT-treated patients being less likely to have a biopsy-to-surgery reduction in PD-L1 expression as compared with those exposed to LC-(C)RT. These findings appear to be supported by preclinical evidence suggesting that hypofractionated radiotherapy may have stronger immunogenic effects, and as such be a better companion treatment for immune checkpoint-based therapies than normofractionated regimens. In this regard, several studies have tested immunotherapy in combination with radiotherapy-containing neoadjuvant regimens, with pathological complete response rates ranging from 22% to 59% [63,64,65,66,67,68,69,70,71,72], and possibly influenced by the differences in radiotherapy schedules and the timing of the administration of immune checkpoint inhibitors [73].

Last but not least, we found an association between higher PD-L1 expression in immune cells from the resection samples and survival outcomes. This is in line with previous studies showing better DFS and/or OS for patients with high PD-L1 expression [40] in tumour cells [44,47,61], immune cells [36,43,48,56,74], or both [36,46] (Supplementary Table S8). Also, the authors of a recent meta-analysis concluded that reduced PD-L1 expression on surgical specimens is an indicator of poor survival, while a higher rate of PD-L1 expression was reported among patients achieving a pathological complete response [75]. This finding is likely to reflect the favourable prognosis of tumours that are either inherently characterised by a rich infiltrate of immune cells or more efficiently targeted because of a stronger treatment-induced immune reaction. Yet, we appreciate that these results require confirmation, especially considering existing reports that mostly support an inverse correlation between PD-L1 expression and outcomes in this disease setting [35,39,41,45,49,50,76].

We acknowledge the limitations of our study, including the retrospective design, the small sample size (due to the inclusion of patients from a single centre), the heterogeneous population and treatments, the pre-defined rounded cut-off values for PD-L1 expression, the exclusion of patients with pathological complete response from the analysis of PD-L1 expression by the TPS and CPS in surgical samples (neither can be calculated in the absence of viable tumour cells), and the risk of random findings due to the lack of statistical adjustment for multiple comparisons. For instance, while the hypothesis of an association between the dynamic changes in PD-L1 and the type of preoperative treatment is intriguing, it is possible that these may be simply explained by the spatial heterogeneity of this biomarker and the well-known limitations of the biopsy sampling [77]. Nevertheless, our findings appear biologically plausible, and would support the current interest for the investigation of immunotherapy-based neoadjuvant strategies whereby immune checkpoint inhibitors are delivered after SCRT, like in the recently reported UNION trial [72]. Also, this study contributes to the literature on PD-L1 in rectal cancer, highlighting the need to standardise analytical procedures, and to better understand the biological bases and clinical significance of post-treatment changes. Finally, it should be recognised that PD-L1 expression is only one small piece of a complex puzzle, and it cannot account for the complexity of the interplay between the tumour and the surrounding microenvironment. Studies looking at all the different biomarkers and cellular components that are involved in the mechanisms of tumour immune response/evasion could be more informative [78].

## 5. Conclusions

In rectal cancer, PD-L1 expression is largely limited to immune cells and tends to reduce in surgical samples, especially among patients treated with neoadjuvant LC-(C)RT. The expression of or increase in PD-L1 expression by the ICS in surgical samples is an independent prognostic factor for patients treated with curative intent. Further analysis of PD-L1 in rectal cancer is warranted, ideally in larger series including patients treated with TNT, which has recently become a new standard of care for locally advanced tumours, and/or with immune checkpoint inhibitors to allow exploring both the prognostic and predictive value of this biomarker.

## Figures and Tables

**Table 1:** Demographics, baseline characteristics, and treatment details.

Patients	100% (83)	Patients	100% (83)
**Sex**		**Preoperative treatment**	
Male	50.6% (42)	No	12% (10)
Female	49.4% (41)	Yes	88% (73)
**Age**		**Type of preoperative treatment**	
Median	63 (30–98)	Radiotherapy	66.3% (55)
<70	68.7% (57)	TNT	19.3% (16)
≥70	31.3% (26)	Chemotherapy	2.4% (2)
**cT stage**		**Type of radiotherapy**	
Ct1	1.2% (1)	SCRT	16.9% (14)
cT2	10.8% (9)	LC-CRT	65.1% (54)
cT3	72.3% (60)	LC-RT	3.6% (3)
cT4	10.8% (9)	NA	14.5% (12)
NA	4.8% (4)	**Type of chemotherapy (for TNT-treated pts)**	
**cN stage**		Induction	4.8% (4)
cN0	19.3% (16)	Consolidation	14.5% (12)
cN1	75.9% (63)		
NA	4.8% (4)		
**cM stage**			
cM0	86.7% (72)		
cM1	13.3% (11)		
**Tumour differentiation**			
G1	22.9% (19)		
G2	56.6% (47)		
G3	3.6% (3)		
NA	16.9% (14)		
**Tumour location**			
Low	37.3% (31)		
Mid	42.2% (35)		
High	20.5% (17)		

Percentages might not total 100 because of rounding. Abbreviations: LC-CRT, long-course chemoradiotherapy; LC-RT, long-course radiotherapy; NA, not available; SCRT, short-course radiotherapy; TNT, total neoadjuvant therapy.

**Table 2:** Variation in PD-L1 expression between paired specimens by neoadjuvant treatment.

	PD-L1 Expression from Biopsy to Resection Specimens								
	TPS			ICS			CPS		
	Decreased	Stable	Increased	Decreased	Stable	Increased	Decreased	Stable	Increased
**Neoadjuvant treatment**									
No	22.2% (2)	77.8% (7)	0% (0)	40% (4)	40% (4)	20% (2)	55.6% (5)	33.3% (3)	11.1% (1)
Yes	14.8% (8)	79.6% (43)	5.6% (3)	41.1% (30)	28.8% (21)	30.1% (22)	31.5% (17)	40.7% (22)	27.8% (15)
*p* value *	0.63	-	1.00	1.00	-	0.72	0.26	-	0.43
**Type of neoadjuvant treatment**									
TNT	11.1% (1)	88.9% (8)	0% (0)	31.2% (5)	37.5% (6)	31.2% (5)	22.2% (2)	33.4% (3)	44.4% (4)
LC-CRT/LC-RT	15.6% (7)	77.8% (35)	6.6% (3)	45.5% (25)	25.4% (14)	29.1% (16)	32.6% (14)	44.2% (19)	23.2% (10)
*p* value *	1.00	-	1.00	0.39	-	1.00	0.70	-	0.23
**Type of radiotherapy**									
SCRT	11.1% (1)	88.9% (8)	0% (0)	14.3% (2)	50% (7)	35.7% (5)	11.1% (1)	66.7% (6)	22.2% (2)
LC-CRT/LC-RT	16.3% (7)	76.7% (33)	7% (3)	49.1% (28)	22.8% (13)	28.1% (16)	34.9% (15)	37.2% (16)	27.9% (12)
*p* value *	1.00	-	1.00	**0.03**	-	0.74	0.24	-	1.00
**Dose of radiotherapy**									
<50 Gy	25% (5)	75% (15)	0% (0)	48.2% (13)	25.9% (7)	25.9% (7)	45% (9)	40% (8)	15% (3)
≥50 Gy	8.7% (2)	78.3% (18)	13% (3)	50% (15)	20% (6)	30% (9)	26.1% (6)	34.8% (8)	39.1% (9)
*p* value *	0.22	-	0.24	1.00	-	0.78	0.22	-	0.10
**Radiotherapy-to-surgery interval**									
<2 months	10% (2)	80% (16)	10% (2)	37% (10)	29.7% (8)	33.3% (9)	25% (5)	55% (11)	20% (4)
≥2 months	18.8% (6)	78.1% (25)	3.1% (1)	45.4% (20)	27.3% (12)	27.3 (12)	34.4% (11)	34.4% (11)	31.2% (10)
*p* value *	0.46	-	0.55	0.62	-	0.60	0.55	-	0.52

* refers to the comparison between decreased versus not decreased (i.e., stable or increased) PD-L1 expression and increased versus not increased (i.e., stable or decreased) PD-L1 expression. Abbreviations: CPS, combined positive score; Gy, gray; ICS: immune-cell score; LC-CRT, long-course chemoradiotherapy; LC-RT, long-course radiotherapy; SCRT, short-course radiotherapy; TNT, total neoadjuvant therapy; TPS, tumour proportion score.

## Data Availability

Data are available upon reasonable request to the corresponding author.

## Supplementary Materials

The following supporting information can be downloaded at: https://www.mdpi.com/article/10.3390/cancers16142606/s1, Table S1. PD-L1 expression ≥1% in biopsy samples by baseline clinico-pathological characteristics; Table S2. PD-L1 expression ≥5% in biopsy samples by baseline clinico-pathological characteristics (no data presented according to TPS due to the lack of PD-L1 ≥5% positive patients); Figure S1. PD-L1 expression ≥5% according to ICS and CPS in biopsy and surgical specimens (no data presented according to TPS due to the virtual lack of PD-L1 ≥5% positive patients); Table S3. Pathological characteristics at surgery; Table S4. Univariable analysis for disease-free survival; Table S5. Multivariable analysis for disease-free survival; Table S6. Univariable analysis for overall survival; Table S7. Multivariable analysis for overall survival; Figure S2. Kaplan-Meier curves for DFS by different PD-L1 scores; Figure S3. Kaplan-Meier curves for DFS according to the change of PD-L1 expression between paired specimens by different scores; Figure S4. Kaplan-Meier curves for OS by different PD-L1 scores; Figure S5. Kaplan-Meier curves for OS according to the change of PD-L1 expression between paired specimens by different scores; Table S8. Selected studies reporting the expression of PD-L1 in locally advanced rectal cancers.
