# Supplementary material for: PD-L1 Expression in Paired Samples of Rectal Cancer
Source: Cancers (Basel). 2024 Jul 21;16(14):2606. doi: 10.3390/cancers16142606 (PMC11275196; doi:10.3390/cancers16142606)
Supplement: Supplementary file 1 [file cancers-16-02606-s001.zip › cancers-3085500-supplementary.pdf]

SUPPLEMENTARY FILES

Table S1. PD-L1 expression ≥1% in biopsy samples by baseline clinico-pathological characteristics

	PD-L1 expression								
	TPS			ICS			CPS		
	Pos	Neg	p value	Pos	Neg	p value	Pos	Neg	p value
**Sex**									
male	6	32	0.92	35	7	0.54	27	11	0.73
female	6	34		32	9		27	13	
**Age**									
≥70 years	1	8	0.43	21	5	0.99	18	6	0.54
<70 years	20	46		46	11		36	18	
**Tumour grading**									
G1	2	17	0.71	15	4	0.74	14	5	1
G2 or G3	8	38		41	9		32	14	
**Tumour location**									
low	2	27	0.19	24	7	0.50	18	11	0.29
mid or high	10	39		45	9		36	13	
**cT stage**									
cT1 or cT2	1	8	1	8	2	0.67	6	3	1
cT3 or cT4	11	54		58	11		46	19	
**cN stage**									
cN0	4	12	0.28	12	4	0.45	12	4	0.76
cN+	8	50		54	9		40	18	

Abbreviations: *TPS, tumour proportion score; ICS: immune-cell score; CPS, combined positive score; Pos, positive; Neg, negative.*

Table S2. PD-L1 expression ≥5% in biopsy samples by baseline clinico-pathological characteristics (no data presented according to TPS due to the lack of PD-L1 ≥5% positive patients)

	**PD-L1 expression**					
	**ICS**			**CPS**		
	Pos	Neg	p value	Pos	Neg	p value
**Sex**						
male	16	26	0.92	10	28	0.89
female	15	26		10	30	
**Age**						
≥70 years	10	16	0.89	8	18	0.93
<70 years	21	36		17	40	
**Tumour grading**						
G1	6	13	0.35	3	16	0.08
G2 or G3	22	28		19	31	
**Tumour location**						
low	10	21	0.46	5	24	0.19
mid or high	21	31		15	34	
**cT stage**						
cT1 or cT2	5	5	0.49	3	6	0.68
cT3 or cT4	25	44		16	49	
**cN stage**						
cN0	8	8	0.27	5	11	0.54
cN+	22	41		14	44	

Abbreviations: *ICS: immune-cell score; CPS, combined positive score; Pos, positive; Neg, negative.*

Figure S1. PD-L1 expression ≥5% according to ICS and CPS in biopsy and surgical specimens (no data presented according to TPS due to the virtual lack of PD-L1 ≥5% positive patients)

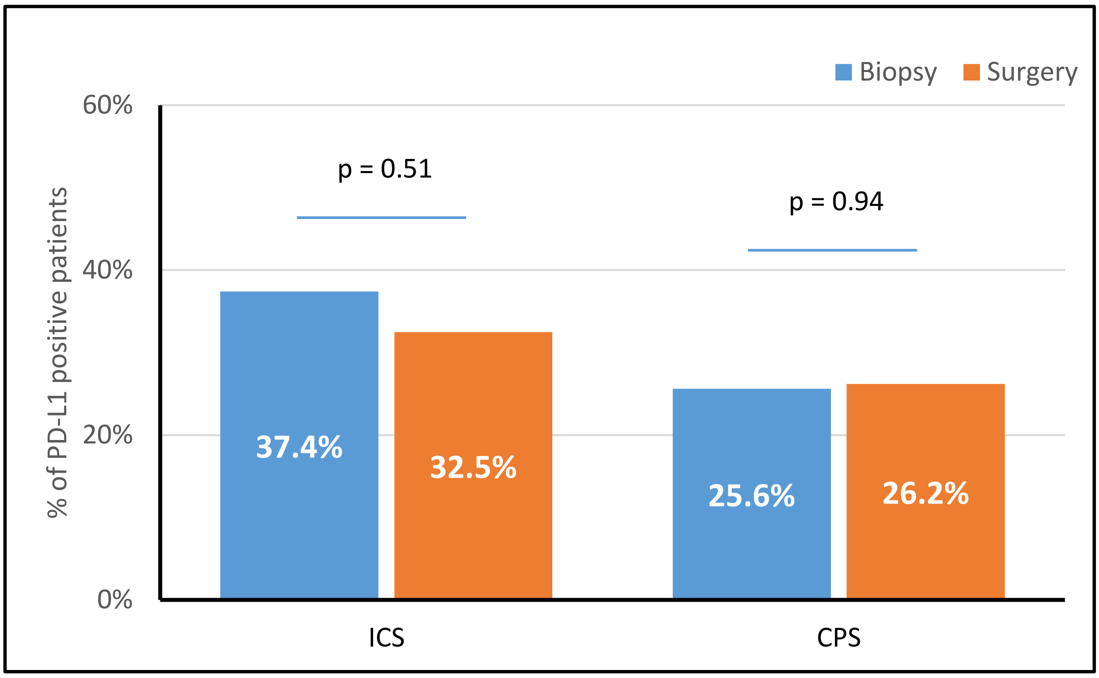

Abbreviations: CPS, combined positive score; ICS: immune-cell score.

Table S3. Pathological characteristics at surgery

**Patients**	100% (83)
**ypT stage**	
ypT0	8.4% (7)
ypTis	3.6% (3)
ypT1	10.8% (9)
ypT2	20.5% (17)
ypT3	48.2% (40)
ypT4	8.4% (7)
**ypN stage**	
ypN0	62.6% (52)
ypN1	22.9% (19)
ypN2	14.5% (12)
**R resection**	
R0	83.1% (69)
R1	13.3% (11)
R2	1.2% (1)
NA	2.4% (2)
**ypVI**	
Positive	28.9% (24)
Negative	65.1% (54)
NA	6% (5)
**ypPNI**	
Positive	31.3% (26)
Negative	61.4% (51)
NA	7.2% (6)
**pCR**	
Yes	8,4% (7)
No	91,6% (76)
**NAR score**	
high	34,9% (29)
intermediate	43,4% (36)
low	16,9% (14)
NA	4,8% (4)

Percentages might not total 100 because of rounding.

Abbreviations: *NA, not available; ypVI, vascular invasion; ypPNI, perineural invasion; NAR; neoadjuvant rectal; pCR, pathological complete response*

Table S4. Univariable analysis for disease-free survival

Variable	HR	p value
Sex (male vs female)	0.89 (0.43-1.9)	0.77
Age (≥70 vs <70 years)	2.3 (1.1-4.9)	**0.031**
Tumour differentiation (biopsy, G2-3 vs G1)	1 (0.43-2.4)	0.99
Tumour location (high/mid vs low)	0.65 (0.31-1.3)	0.24
cT stage (cT3-4 vs cT1-2)	1.2 (0.4-3.3)	0.79
cN stage (cN+ vs cN0)	0.64 (0.28-1.5)	0.29
Preoperative treatment (yes vs no)	0.79 (0.27-2.3)	0.66
Type of preoperative treatment (TNT vs RT only)	1.4 (0.46-4.3)	0.55
Type of radiotherapy (LC-(C)RT vs SCRT)	0.57 (0.2-1.6)	0.29
Radiotherapy dose (≥50 vs <50 Gy)	0.46 (0.18-1.2)	0.11
Radiotherapy-to-surgery interval (≥2 vs <2 months)	1 (0.45-2.2)	0.99
ypT stage (ypT3-4 vs ypT0-2)	2.1 (0.98-4.7)	**0.057**
ypN stage (ypN1-2 vs ypN0)	1.8 (0.84-3.9)	0.13
Tumour differentiation (surgery, G2-3 vs G1)	2.5 (0.85-7.3)	**0.096**
Resection (R1-2 vs R0)	2 (0.79-5)	0.15
Pathological complete response (yes vs no)	3.8e-09 (0-Inf)	1
YpVI (yes vs no)	3.5 (1.6-7.7)	**0.002**
YpPNI (yes vs no)	5.7 (2.4-14)	**6.60e-05**
NAR score (low/intermediate vs high)	0.55 (0.26-1.2)	0.12
Biopsy PD-L1 TPS (≥1% vs <1%)	0.27 (0.036-2)	0.2
Surgery PD-L1 TPS (≥1% vs <1%)	0.71 (0.095-5.3)	0.74
Biopsy PD-L1 ICS (≥1% vs <1%)	1 (0.43-2.4)	0.97
Biopsy PD-L1 ICS (≥5% vs <5%)	0.77 (0.27-2.2)	0.64
Surgery PD-L1 ICS (≥1% vs <1%)	0.68 (0.32-1.4)	0.32
Surgery PD-L1 ICS (≥5% vs <5%)	0.34 (0.13-0.89)	**0.029**
Biopsy PD-L1 CPS (≥1% <1%)	1.1 (0.49-2.4)	0.86
Biopsy PD-L1 CPS (≥5% vs <5%)	0.44 (0.15-1.3)	0.13
Surgery PD-L1 CPS (≥1% vs <1%)	0.81 (0.37-1.8)	0.59
Surgery PD-L1 CPS (≥5% vs <5%)	0.32 (0.1-1.1)	**0.067**
Biopsy-to-surgery increase of PD-L1 TPS	0.72 (0.097-5.4)	0.75
Biopsy-to-surgery decrease of PD-L1 TPS	0.25 (0.033-1.8)	0.17
Biopsy-to-surgery increase of PD-L1 IC	0.44 (0.17-1.2)	**0.095**
Biopsy-to-surgery decrease of PD-L1 IC	0.99 (0.47-2.1)	0.97
Biopsy-to-surgery increase of PD-L1 CPS	0.52 (0.18-1.5)	0.23
Biopsy-to-surgery decrease of PD-L1 CPS	0.75 (0.33-1.7)	0.49

Abbreviations: *CPS, combined positive score; Gy, gray; HR, hazard ratio; ICS: immune-cell score; LC-(C)RT, long-course (chemo)radiotherapy; NAR, neoadjuvant rectal; RT, radiotherapy; SCRT, short-course radiotherapy; TNT, total neoadjuvant therapy; TPS, tumour proportion score; ypPNI, perineural invasion; ypVI, vascular invasion.*

Table S5. Multivariable analysis for disease-free survival

Multivariable model for surgical PD-L1 ICS ≥5%		
**Variable**	**HR**	**p value**
Age	4.39 (1.48-13.06)	**0.008**
ypT stage	1.74 (0.6-5.02)	0.31
Tumour differentiation (surgery)	1.91 (0.51-7.16)	0.34
ypVI	0.9 (0.26-3.1)	0.86
ypPNI	6.78 (1.87-24.55)	**0.004**
Surgery PD-L1 ICS (≥5%)	0.17 (0.04-0.77)	**0.02**
Multivariable model for surgical PD-L1 CPS ≥5%		
**Variable**	**HR**	**p value**
Age	2.76 (0.88-8.7)	0.08
ypT stage	1.26 (0.43-3.65)	0.67
Tumour differentiation (surgery)	2.17 (0.43-10.86)	0.35
ypVI	1.88 (0.55-6.43)	0.32
ypPNI	4.92 (1.33-18.16)	**0.02**
Surgery PD-L1 CPS (≥5%)	0.44 (0.11-1.78)	0.25
Multivariable model for biopsy-to-surgery increase of PD-L1 by IC		
**Variable**	**HR**	**p value**
Age	4.54 (1.52-13.56)	**0.007**
ypT stage	1.69 (0.58-4.89)	0.34
Tumour differentiation (surgery)	1.97 (0.52-7.44)	0.32
ypVI	0.97 (0.28-3.44)	0.97
ypPNI	6.95 (1.87-25.76)	**0.004**
Biopsy-to-surgery increase of PD-L1 by ICS	0.19 (0.04-0.89)	**0.04**

Abbreviations: *CPS, combined positive score; HR, hazard ratio; ICS: immune-cell score; ypPNI, perineural invasion; ypVI, vascular invasion.*

Table S6. Univariable analysis for overall survival

Variable	HR	p value
Sex (male vs female)	0.59 (0.25-1.4)	0.23
Age (≥70 vs <70 years)	3.5 (1.5-8.2)	**0.005**
Tumour differentiation (biopsy, G2-3 vs G1)	0.73 (0.28-1.9)	0.52
Tumour location (high/mid vs low)	0.6 (0.26-1.4)	0.23
cT stage (cT3-4 vs cT1-2)	1.1 (0.33-3.9)	0.84
cN stage (cN+ vs cN0)	0.79 (0.31-2)	0.63
Preoperative treatment (yes vs no)	0.48 (0.16-1.5)	0.20
Type of preoperative treatment (TNT vs RT only)	2.2 (0.42-11)	0.35
Type of radiotherapy (LC-(C)RT vs SCRT)	0.31 (0.07-1.4)	0.12
Radiotherapy dose (≥50 vs <50 Gy)	0.65 (0.21-2.1)	0.47
Radiotherapy-to-surgery interval (≥2 vs <2 months)	1.1 (0.41-2.7)	0.90
ypT stage (ypT3-4 vs ypT0-2)	1.7 (0.71-4.1)	0.23
ypN stage (ypN1-2 vs ypN0)	1.5 (0.61-3.7)	0.38
Tumour differentiation (surgery, G2-3 vs G1)	2.6 (0.75-9.4)	0.13
Resection (R1-2 vs R0)	2.6 (0.92-7.2)	**0.072**
Pathological complete response (yes vs no)	3.8e+09 (0-Inf)	1.00
YpVI (yes vs no)	2.5 (1-6.1)	**0.043**
YpPNI (yes vs no)	6.7 (2.3-19)	**<0.001**
NAR score (low/intermediate vs high)	0.68 (0.28-1.7)	0.40
Biopsy PD-L1 TPS (≥1% vs <1%)	0.25 (0.03-1.9)	0.18
Surgery PD-L1 TPS (≥1% vs <1%)	1.1 (0-Inf)	0.59
Biopsy PD-L1 ICS (≥1% vs <1%)	0.73 (0.29-1.8)	0.50
Biopsy PD-L1 ICS (≥5% vs <5%)	0.75 (0.22-2.6)	0.65
Surgery PD-L1 ICS (≥1% vs <1%)	0.46 (0.19-1.1)	**0.084**
Surgery PD-L1 ICS (≥5% vs <5%)	0.28 (0.08-0.95)	**0.042**
Biopsy PD-L1 CPS (≥1% <1%)	0.93 (0.4-2.2)	0.87
Biopsy PD-L1 CPS (≥5% vs <5%)	0.4 (0.12-1.4)	0.15
Surgery PD-L1 CPS (≥1% vs <1%)	0.67 (0.27-1.7)	0.40
Surgery PD-L1 CPS (≥5% vs <5%)	0.14 (0.02-1.1)	**0.059**
Biopsy-to-surgery increase of PD-L1 TPS	3 (0.18-48)	0.44
Biopsy-to-surgery decrease of PD-L1 TPS	0.34 (0.021-5.5)	0.44
Biopsy-to-surgery increase of PD-L1 IC	0.34 (0.099-1.2)	**0.082**
Biopsy-to-surgery decrease of PD-L1 IC	1.1 (0.48-2.6)	0.8
Biopsy-to-surgery increase of PD-L1 CPS	0.5 (0.15-1.7)	0.26
Biopsy-to-surgery decrease of PD-L1 CPS	0.97 (0.41-2.3)	0.95

Abbreviations: *CPS, combined positive score; Gy, gray; HR, hazard ratio; ICS: immune-cell score; LC-(C)RT, long-course (chemo)radiotherapy; NAR, neoadjuvant rectal; RT, radiotherapy; SCRT, short-course radiotherapy; TNT, total neoadjuvant therapy; TPS, tumour proportion score; ypPNI, perineural invasion; ypVI, vascular invasion.*

Table S7. Multivariable analysis for overall survival

Multivariable model for surgical PD-L1 ICS ≥1%		
**Variable**	**HR**	**p value**
Age	7.35 (2.11-25.55)	**0.002**
Resection	1.84 (0.48-7.00)	0.37
ypVI	0.95 (0.32-2.79)	0.92
ypPNI	7.9 (2.1-29.78)	**0.002**
Surgery PD-L1 ICS ≥1%	0.28 (0.08-0.97)	**0.04**
Multivariable model for surgical PD-L1 ICS ≥5%		
**Variable**	**HR**	**p value**
Age	5.36 (1.81-15.93)	**0.002**
Resection	1.49 (0.4-5.64)	0.55
ypVI	0.51 (0.14-1.85)	0.31
ypPNI	9.4 (2.41-36.92)	**0.001**
Surgery PD-L1 ICS ≥5%	0.19 (0.04-0.88)	**0.03**
Multivariable model for surgical PD-L1 CPS ≥5%		
**Variable**	**HR**	**p value**
Age	3.42 (1.27-9.21)	**0.015**
Resection	1.72 (0.46-6.53)	0.42
ypVI	0.81 (0.28-2.4)	0.71
ypPNI	4.9 (1.33-18.04)	**0.02**
Surgery PD-L1 CPS ≥5%	0.21 (0.03-1.71)	0.14
Multivariable model for biopsy-to-surgery increase of PD-L1 by IC		
**Variable**	**HR**	**p value**
Age	5.47 (1.82-16.37)	**0.002**
Resection	1.50 (0.40-5.7)	0.55
ypVI	0.51 (0.14-1.89)	0.32
ypPNI	9.78 (2.48-38.63)	**0.001**
Biopsy-to-surgery increase of PD-L1 by IC	0.20 (0.04-0.93)	**0.04**

Abbreviations: *CPS, combined positive score; HR, hazard ratio; ICS: immune-cell score; ypPNI, perineural invasion; ypVI, vascular invasion.*

Figure S2. Kaplan-Meier curves for DFS by different PD-L1 scores

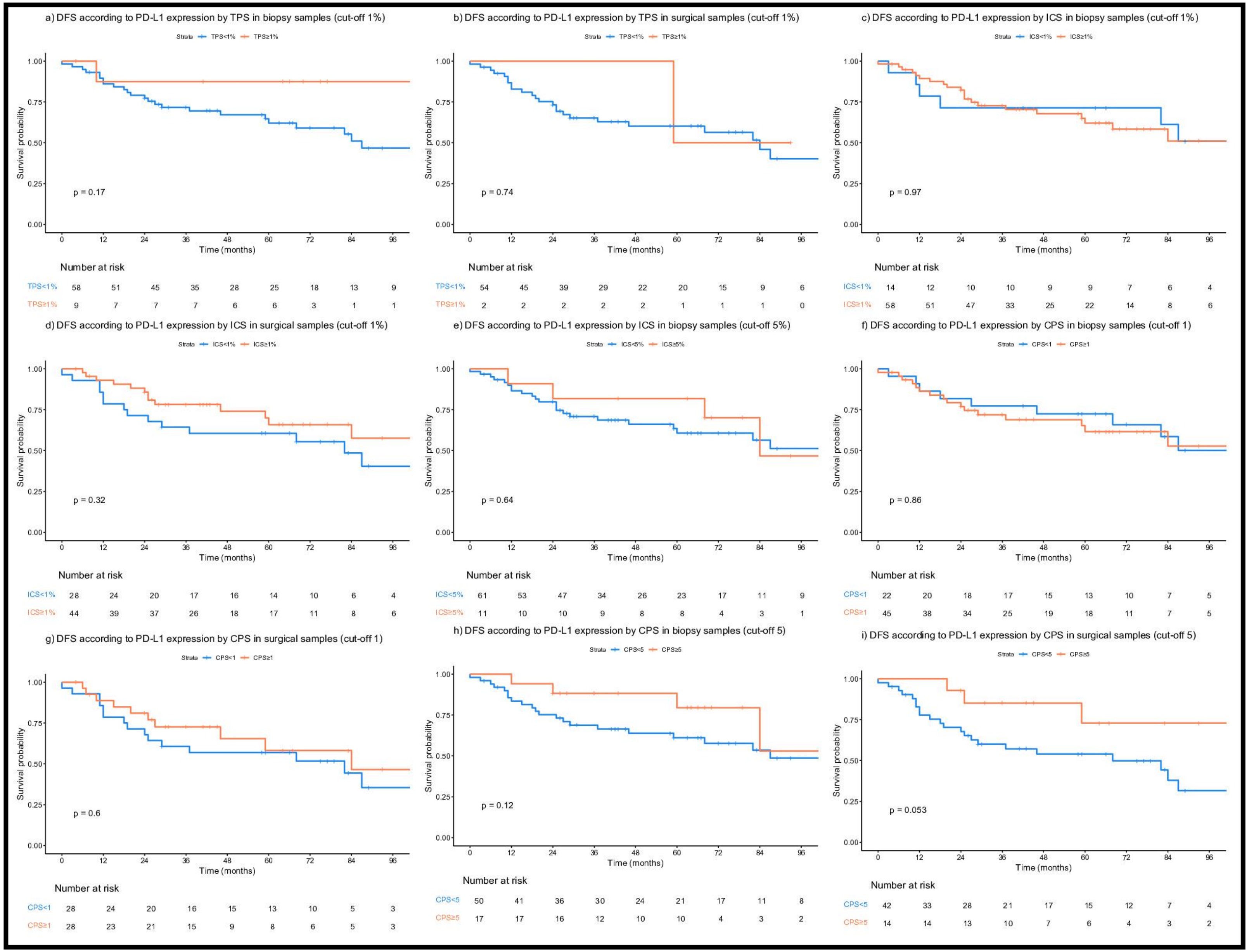

***Abbreviations**: CPS, combined positive score; DFS: disease-free survival; ICS: immune cell score; PD-L1: programmed death-ligand 1; TPS, tumour proportion score.*

Figure S3. Kaplan-Meier curves for DFS according to the change of PD-L1 expression between paired specimens by different scores

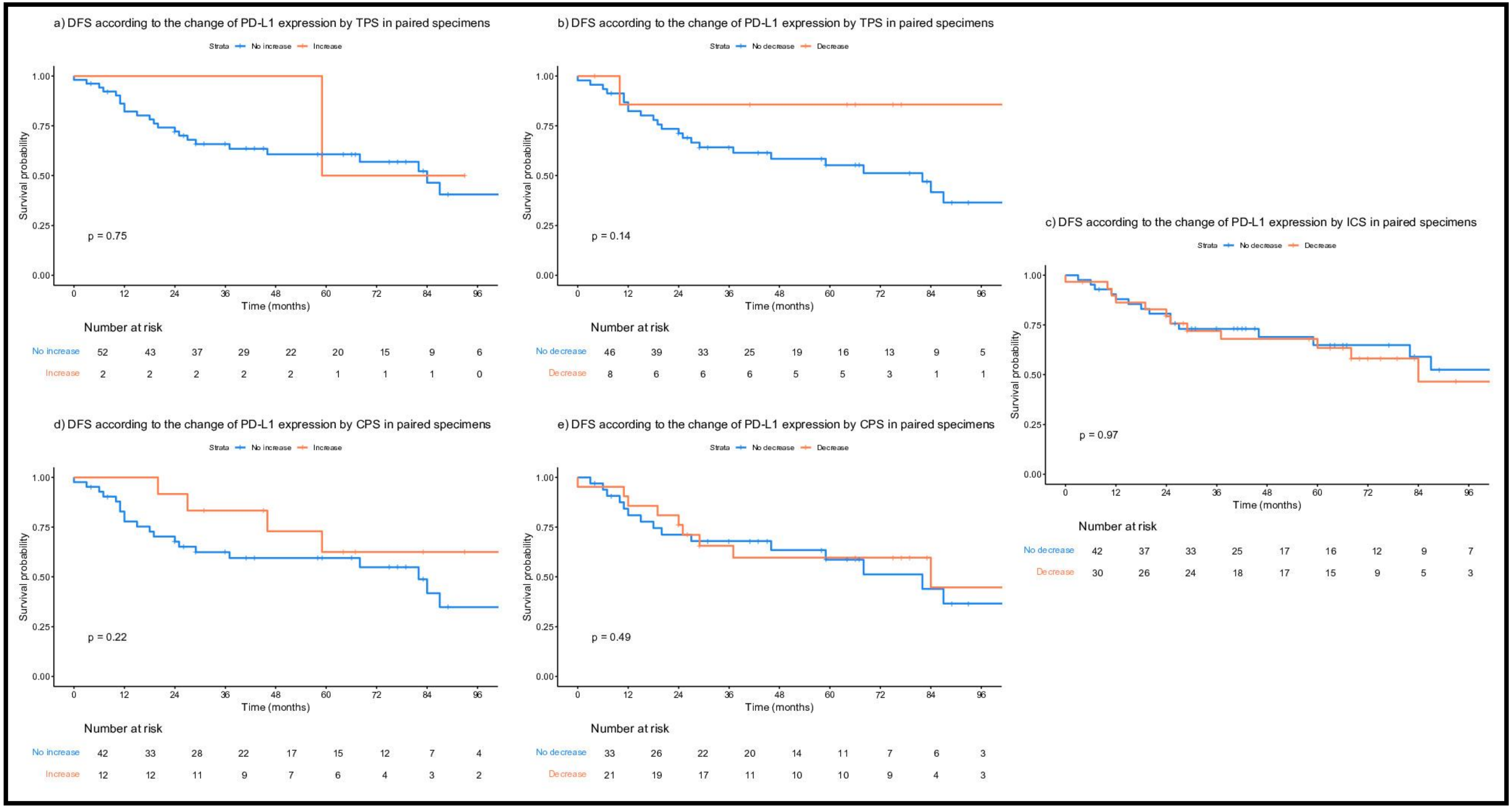

***Abbreviations**: CPS, combined positive score; DFS: disease-free survival; ICS: immune cell score; PD-L1: programmed death-ligand 1; TPS, tumour proportion score.*

Figure S4. Kaplan-Meier curves for OS by different PD-L1 scores

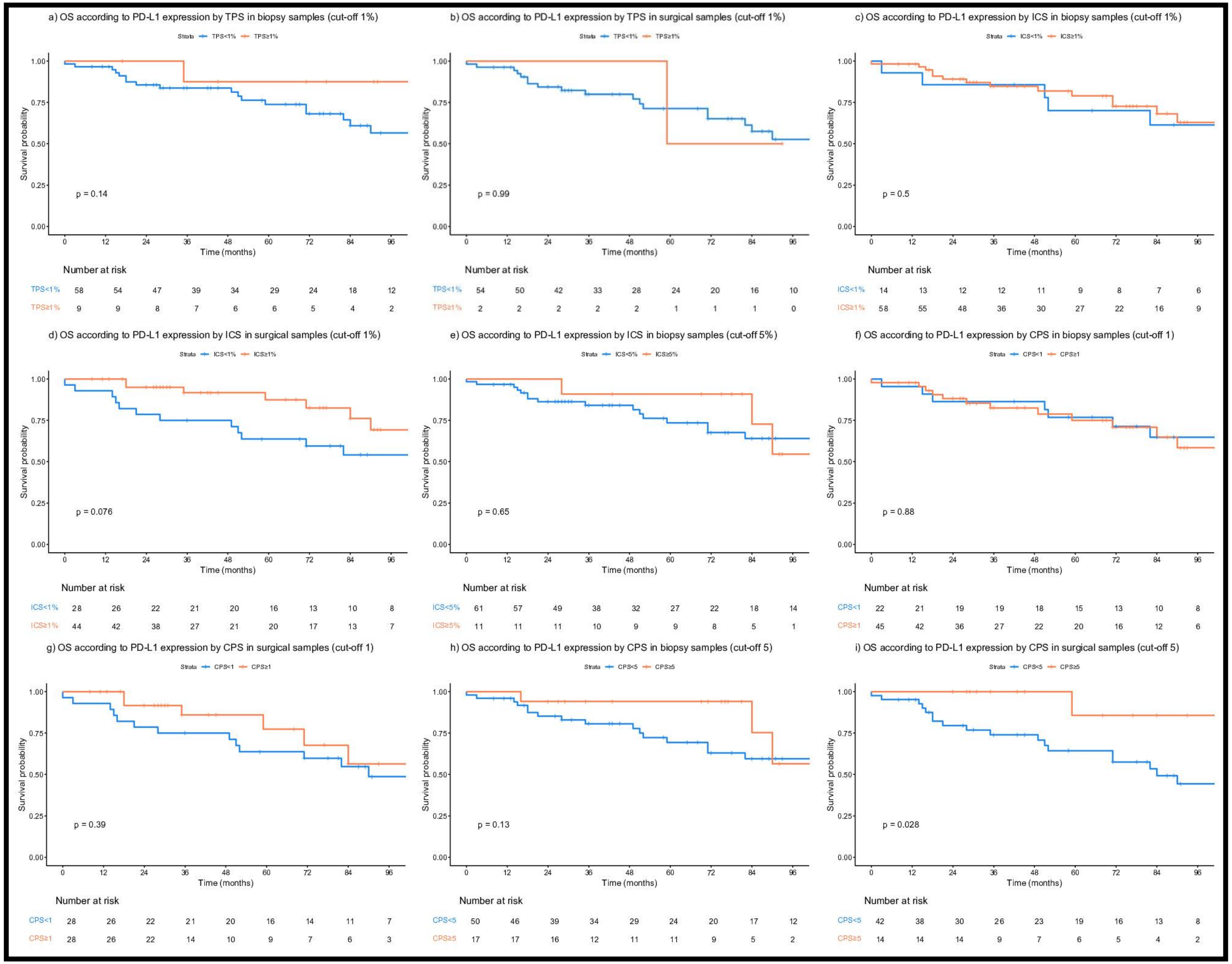

***Abbreviations**: CPS, combined positive score; ICS: immune cell score; OS: overall survival; PD-L1: programmed death-ligand 1; TPS, tumour proportion score.*

Figure S5. Kaplan-Meier curves for OS according to the change of PD-L1 expression between paired specimens by different scores

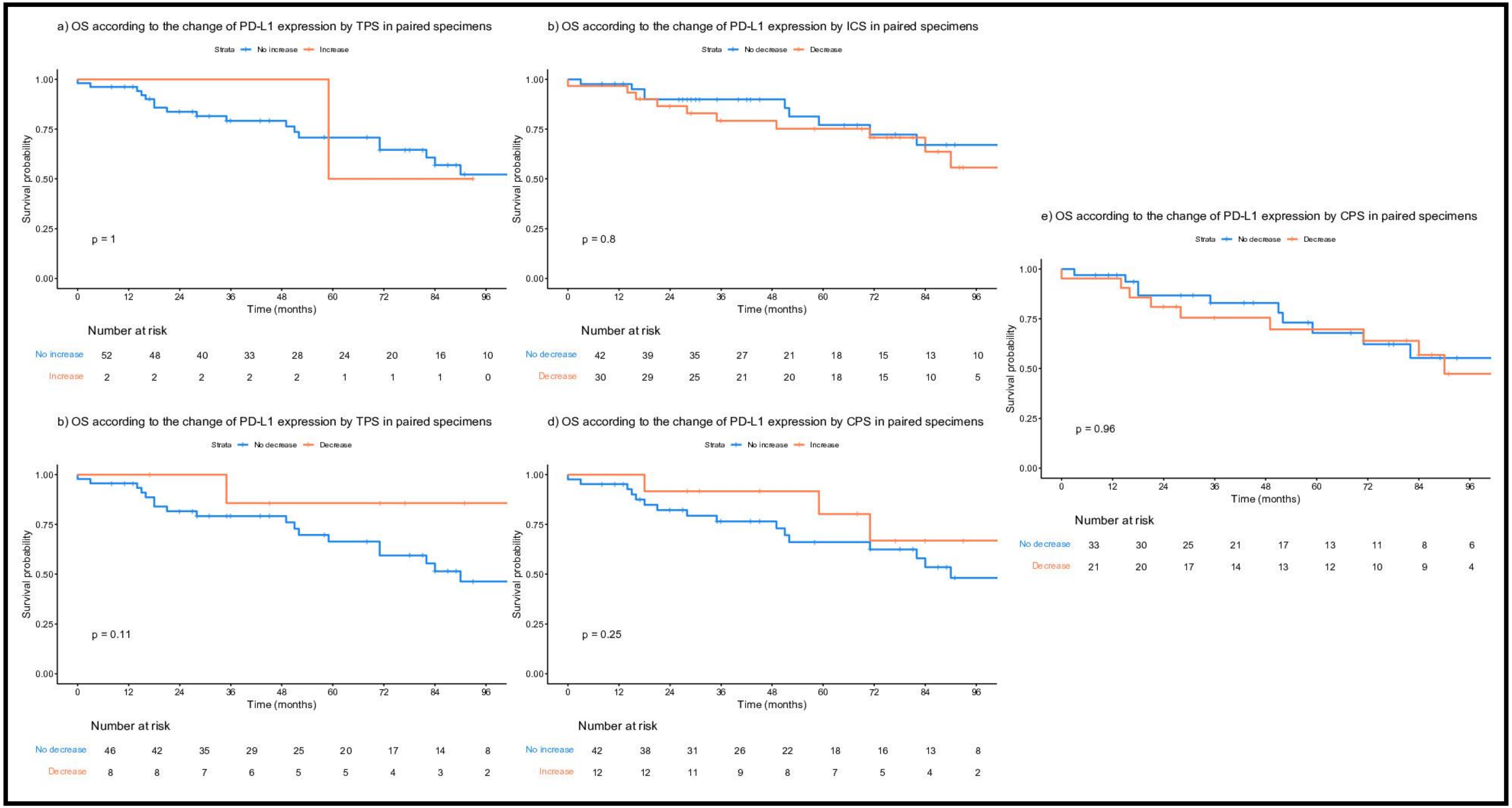

***Abbreviations**: CPS, combined positive score; ICS: immune cell score; OS: overall survival; PD-L1: programmed death-ligand 1; TPS, tumour proportion score.*

Table S8. Selected studies reporting the expression of PD-L1 in locally advanced rectal cancers

Study	N pts	PD-L1 Ab clone	PD-L1 score	PD-L1 cut-off or assessment method	Neoadjuvant therapy	PD-L1 expression (pre-treatment biopsy)*	PD-L1 expression (surgery)*	Variation of PD-L1 expression between biopsy and surgery	Prognostic value
Teng et al (2015)	62 (LCRT), 31 (surgery)	M4424	TC, IC	Semiquantitative	FP-based LCRT (details of CT not available)	Pre-LCRT: 1.6% (TC), 4.8% (IC)<br>Pre-surgery: 0% TC, 3.2% (IC)	Post-LCRT: 6.4% (TC), 3.2% (IC)<br>Post-surgery alone: 0% TC, 6.4% (IC)	No significant variation	No prognostic role
Saigusa et al (2016)	90	27A2	TC	Semiquantitative	LCRT (52%) or SCRT (48%)	NA	40%	NA	High PD-L1 expression after LCRT was a negative prognostic factor for RFS and OS
Hecht et al (2016)	199 (103 with biopsy samples, 159 with surgical samples, 63 with paired samples)	E1L3N	TC, IC	Semiquantitative (median cut-off)	LCRT with 5-FU (52.2%), 5-FU + oxaliplatin (38.4%), or other (8.8%)	2.1% (TC), 14% (ICs) of cells	7.8% (TC, central tumour), 9.3% (TC, invasive front), 15% (IC, central tumour), 12% (IC, invasive front) of cells	PD-L1 intensity not significantly increased in TC, significantly decreased in IC	Low PD-L1 expression in TC and IC in pre-CRT specimens as well as in the invasive front of post-CRT specimens were negative prognostic factors for OS
Jomrich et al (2016)	29	E1L3N (for biopsy samples), 5H1 (for surgical samples)	TC, IC	Semiquantitative	LCRT	0%	0% (TC), 17.24 % (IC)	Increase in IC	NA
Richter et al (2017)	25	28-8	TC	≥1%	LCRT	0%	0%	-	-

Shao et al (2017)	68	SP142	TC, IC	≥1% (TC), ≥10% (IC)	SCRT (58.8%), LRT (41.2%), CT (29.4%)	NA	10.3% (TC), 25% (IC)	PD-L1 expression in TC significantly correlated with SCRT	PD-L1 expression in TC after RT was a negative prognostic factor for LRFS
Huang et al (2017)	87 (73 with paired samples)	NR	NR	NR	LCRT (capecitabine or tegafur/uracil)	54%	64%	Increase	PD-L1 expression after LCRT was a positive prognostic factor for OS and DFS
Lim et al (2017)	123	Q9NZQ7	TC	Semiquantitative (median cut-off)	LCRT	Median H score: 0	Median H score: 100	Significant increase	High level of PD-L1 expression in biopsy samples was a negative prognostic factor for OS and DFI
Ogura et al (2017)	287	205921	TC, IC	≥5%	LCRT	1% (TC), 31.7% (IC)	1% (TC), 49.2% (IC)	Significant increase in IC	High levels of PD-L1 expression in IC after LCRT were associated with tumour response and DFS
Chiang et al (2018)	104	205921	TC	≥5%	LCRT	51%	64%	Increase	PD-L1 expression after LCRT was a positive prognostic factor for OS and DFS
Yamashita et al (2018)	68 (44 LCRT, 24 upfront surgery)	NR	TC, IC	NR	LCRT	0% (TC), NR (IC)	11% (TC) after CRT	Significant increase in IC after LCRT, no significant increase in TC after LCRT	PD-L1 expression in IC was a negative prognostic factor for OS
									Increase of PD-L1 expression after

Bae et al (2019)	24	NR	NR	NR	LCRT	16.7%	45%	Significant increase at the tumour margins	LCRT was associated with reduced response to LCRT
Boustani et al (2020)	74	QR1	Any cell (TC or IC)	Semiquantitative	FP-based LCRT (with or without platinum agents) (63.5%), SCRT (36.5%)	Median rate of PD-L1+ cells: 15%	Median rate of PD-L1+ cells: 50%	Significant increase. (regardless of RT fractionation)	Median OS and PFS were higher in pre-LCRT biopsies with >8% PD-L1+ cells. High levels of PD-L1 expression in both biopsy and surgical samples were a positive prognostic factor for OS and PFS.
Huemer et al (2020)	72	22C3	TPS, CPS, ICS	>1% and median cut-off	LCRT with FP (40%), or FP + oxaliplatin (60%)	93% (TPS), 97 (CPS), 97% (ICS) Median expression: 4% (TPS), 18.5 (CPS), 13% (ICS)	Median expression: 0% (TPS), 3 (CPS), 3% (IC)	Significant decrease, regardless of the CT backbone	PD-L1 expression ≤1% by TPS prior to and after CRT was a negative prognostic factor for OS
Feng et al (2022)	103	MIH1	TPS, ICS, CPS	Semiquantitative (mean cut-off)	LCRT	2.2% (CPS), 1.9 (TPS), 0.7% (IC)	2.2% (CPS), 1.38 (TPS), 1.6% (IC)	Significant increase (ICS)	Pre-CRT TPS and post-CRT CPS were positive prognostic factor for DFS.
Takahashi et al (2022)	109 (LCRT), 34 (upfront surgery)	28-8	TC, IC	≥5% (TC), ≥30% (IC)	LCRT with S1 + irinotecan	0% in IC, NR for TC	Upfront surgery: 2.9% (TC), NR for IC<br><br>Post-LCRT: 2.4% (TC), NR for IC	NR	High PD-L1 expression in IC after LCRT was a negative prognostic factor for OS
`Lim et al (2022)	165	E1L3N	TC, IC	Semiquantitative (median cut-off)	LCRT	NA	Median H score: 140 (TC), 120 (IC)	NA	Low PD-L1 expression in IC after LCRT, and high PD-L1

									expression in TC after LCRT were negative prognostic factors for DFS
Baretti et al (2022)	62 (LCRT), 17 (upfront surgery)	SP142	TC, IC interface	NR	LCRT	NA	Upfront surgery: 0% (TC), 64.7% (IC), 76.5% (IF)	No significant variation	NA

* Percentages of PD-L1 expression refer to patients unless otherwise specified

***Abbreviations**: Ab, antibody; CPS, combined positive score; CRC, colorectal cancer; CT, chemotherapy; DFI, disease-free interval; DFS: disease-free survival; FP, fluoropyrimidine; fr, fractions; Gy, Gray; ICS: immune cell score; LCRT: long-course chemoradiotherapy*; LRFS, local recurrence-free survival; LRT, long-course radiotherapy; N, number; NA, not assessed; NR, not reported; OS: overall survival; pCR, pathological complete response; PD-L1: programmed death-ligand 1; pts, patients; PFS, progression-free survival; RC, rectal cancer; RFS: recurrence-free survival; SCRT, short-course radiotherapy; TPS, tumour proportion score.*
